# Rivaroxaban and Piperacillin–Tazobactam: Case of Massive Gastrointestinal Bleeding

**DOI:** 10.1155/carm/6053487

**Published:** 2026-01-31

**Authors:** Abbas Mohammadi, Masoud Bitarafan, Korosh Hamed Saedian, Hossein Akhondi

**Affiliations:** ^1^ Department of Internal Medicine, Valley Health System, Las Vegas, Nevada, USA

**Keywords:** anticoagulation, case report, drug–drug interactions, gastrointestinal bleeding, piperacillin–tazobactam, rivaroxaban

## Abstract

Anticoagulated patients are at increased risk of complications from drug interactions, including with antibiotics. Piperacillin–tazobactam (PTZ) has been associated with coagulation disruptions, potentially leading to severe bleeding when combined with anticoagulants like rivaroxaban. This report describes the first known case of massive gastrointestinal (GI) bleeding potentially associated with PTZ in a rivaroxaban‐treated patient, successfully managed by discontinuing both drugs. We present a 52‐year‐old male with deep vein thrombosis on rivaroxaban, admitted for severe left leg cellulitis. Shortly after starting PTZ, he experienced massive GI bleeding with hematochezia, hematemesis, and syncope, requiring urgent intervention, cessation of both medications, and esophagogastroduodenoscopy (EGD), which revealed minor erosions. The temporal relationship suggests a possible PTZ‐induced hemostasis disruption or interaction with rivaroxaban. This case highlights the need for research into PTZ–anticoagulant interactions and emphasizes vigilant monitoring and timely management to reduce life‐threatening bleeding risks in anticoagulated patients.

## 1. Introduction

The integration of anticoagulants, such as rivaroxaban, into the management of conditions like deep vein thrombosis (DVT) has transformed treatment strategies by offering predictable effects, minimal interactions, and eliminating the need for routine monitoring seen with warfarin [1–3]. Despite its advantages, rivaroxaban’s safety profile may be compromised when combined with certain medications. This is particularly relevant for antibiotics, a frequently coprescribed drug class. It is well documented that the risk of bleeding increases when direct oral anticoagulants (DOACs) like rivaroxaban are combined with antibiotics such as macrolides and fluoroquinolones, often due to pharmacokinetic interactions involving the P‐glycoprotein and CYP3A4 pathways [4]. In contrast, the risk associated with the broad‐spectrum antibiotic piperacillin–tazobactam (PTZ) is less characterized and may involve distinct, pharmacodynamic mechanisms such as impaired hemostasis [5–7].

PTZ is frequently used to treat severe bacterial infections, but it has been implicated in coagulation disturbances. Although the precise mechanisms remain unclear, PTZ is thought to impair platelet function and influence the synthesis of clotting factors, potentially increasing bleeding risk when coadministered with anticoagulants [5–8]. Some reports suggest PTZ suppresses intestinal flora, reducing vitamin K2 production—a critical component for activating clotting factors II, VII, IX, and X and prothrombin [9, 10]. These effects, though subtle in isolation, may have profound clinical implications in patients receiving anticoagulant therapy [9].

Evidence linking PTZ to coagulation disturbances includes findings of reduced platelet aggregation, prolonged bleeding times, and rare cases of significant hemorrhagic events [9, 10]. For example, Bower et al. reported intracranial hemorrhages attributed to PTZ‐induced platelet dysfunction rather than thrombocytopenia [5]. Discontinuation of PTZ normalized platelet function, emphasizing the need for further investigation into its hemostatic effects [5]. While such findings have largely focused on laboratory data, they raise concerns about the potential for life‐threatening bleeding, particularly in patients on DOACs.

This case report presents the first documented instance of severe gastrointestinal (GI) bleeding in a patient who had recently started PTZ administration. By reviewing the clinical course, diagnostic findings, and treatment responses, we aim to elucidate the underlying mechanisms of this drug side effect and propose strategies to optimize care for patients navigating the complex intersection of cardiovascular disease, infection, and anticoagulation therapy.

## 2. Case Presentation

A 52‐year‐old white man with a significant medical history, including DVT managed with rivaroxaban 20 mg orally once daily (a full therapeutic dose for DVT treatment) for 3 months, morbid obesity, and a history of smoking, presented to the emergency department. The patient denied any prior history of GI bleeding, peptic ulcer disease, or liver cirrhosis. He also confirmed no use of other anticoagulant or antiplatelet medications in the 3 months preceding this admission. He reported a sudden onset of these symptoms a few days prior, accompanied by loss of appetite, chills, and rigors. The patient denied abdominal discomfort, urinary complaints, chest pain, or respiratory distress and disclosed smoking marijuana but denied alcohol or other illicit substance use.

On presentation, vital signs were stable. Notably, laboratory investigations revealed a markedly elevated white blood cell (WBC) count of 29.75 × 10^9^/L, suggesting a significant inflammatory or infectious process. Hemoglobin (Hb) was 14.3 g/dL, and platelet count was 245 × 10^9^/L. The C‐reactive protein (CRP) level was elevated at 28.5 mg/L. However, coagulation profiles, renal function tests (BUN and creatinine), electrolytes, and liver function tests (LFTs) were all within normal limits (Table 1). On physical examination, the left foot was erythematous and warm. While Doppler ultrasound ruled out recurrent DVT, computed tomography (CT) scans showed phlegmonous changes and extensive subcutaneous edema consistent with cellulitis. The Infectious Disease service was consulted, and empiric broad‐spectrum antibiotics were initiated (PTZ at a dose of 4.5 g intravenously every 8 h, vancomycin, and clindamycin) for severe cellulitis. This was despite a CURB‐65 score of 0, due to concerning systemic symptoms and a markedly elevated WBC count.

**TABLE 1:** Patient’s clinical course and laboratory data.

Day	Vital signs	Laboratory findings	Normal range	Imaging/procedures	Interventions
Day 1	Temp: 36.9°C, pulse: 75 bpm, BP: 147/73 mmHg	WBC: 29.75 × 10^9^/L, Hb: 14.3 g/dL, PLT: 245 × 10^9^/L, CRP: 28.5 mg/L, PT/INR: normal, fibrinogen: normal	WBC: 4.0–11.0 × 10^9^/L Hb: 13.5–17.5 g/dL Platelet Count: 150–450 × 10^9^/L CRP: < 10.0 mg/L	CT: phlegmonous changes, subcutaneous edema	Empirical antibiotics: piperacillin–tazobactam, vancomycin, clindamycin
Day 2 (AM)	Temp: 36.7°C ⟶ 38°C, pulse: 83 ⟶ 102 bpm, BP: 124/62 ⟶ 95/58 mmHg	Hb: 11.9 g/dL ⟶ 8.7 g/dL, PT: 13.6 s, INR: 1.3, fibrinogen: 628 mg/dL, K: 2.9 mEq/L		Ultrasound: negative for DVT	Discontinued rivaroxaban and all prior antibiotics. Added daptomycin. Two large‐bore IV lines established. Reserved 2 units of packed RBCs. Gastric/esophageal bleeding managed with fluids and transfusions.
Day 2 (PM)	—	—		EGD: active oozing antral ulcer (8 mm), old blood in duodenum	Treated ulcer with irrigation, suction, and hemostasis. Initiated high‐dose IV pantoprazole (72 h) followed by maintenance.
Day 3	Stable vitals	Hb improving, other labs are normal		—	Downgraded to general medical floor. Resumed oral intake without complications.

*Note:* Legends: Vitals: Temp (temperature); pulse (heart rate, HR). Laboratory findings: WBC (white blood cell count, × 10^9^/L), Hb (hemoglobin, g/dL), PLT (platelet count, × 10^9^/L), CRP (C‐reactive protein, mg/L), PT (prothrombin time, seconds), K (potassium, mEq/L), and fibrinogen (mg/dL). EGD (esophagogastroduodenoscopy), IV (intravenous).

Abbreviations: BP, blood pressure; CT, computed tomography; DVT, deep vein thrombosis; INR, international normalized ratio; RBC, red blood cell transfusion.

**FIGURE 1:**
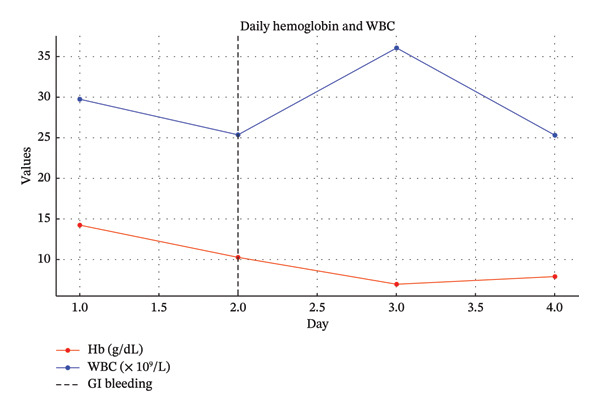
Trends in hemoglobin and white blood cell count in our patient. This figure illustrates the daily variations in hemoglobin (Hb) and white blood cell count (WBC) over a 5‐day treatment period. Hemoglobin levels, represented in red, show a decline following the second day, which coincides with the gastrointestinal bleeding events indicated by the dashed line. This decline likely reflects the blood loss associated with the bleeding events. Conversely, the white blood cell count, depicted in blue, demonstrates fluctuations indicative of the patient’s inflammatory response to infection and subsequent medical interventions. The dashed line on Day 2 highlights the critical intervention point where gastrointestinal bleeding was observed, necessitating changes in the treatment regimen, including the cessation of piperacillin/tazobactam. These trends provide insights into the patient’s response to the medical crisis and the effectiveness of the therapeutic measures implemented.

On the second hospital day, the patient experienced a syncopal episode early in the morning, accompanied by hematochezia and hematemesis. He reported bright red blood per rectum, hematemesis, severe weakness, and generalized body pain. Vital signs showed a rise in temperature from 36.7°C to 38°C, an escalation in pulse rate from 83 to 102 beats per minute, and a significant drop in blood pressure from 124/62 to 95/58 mmHg. Laboratory investigations revealed a sharp decline in Hb to 8.7 g/dL, as shown in Figure 1. Notably, the platelet count was 266 × 10^9^/L, which was within the normal range. Coagulation parameters showed an increase in prothrombin time (PT) to 13.6 s and international normalized ratio (INR) to 1.3, along with a fibrinogen level elevated to 628 mg/dL. Additionally, potassium levels dropped to 2.9 mEq/L.

The patient reported mild generalized abdominal pain and a sudden loss of appetite. Gastroenterology was urgently consulted. Rivaroxaban, last administered the previous day, was discontinued following the bleeding presentation. The patient denied any prior GI bleeding symptoms, such as melena, hematochezia, or hematemesis, and had never undergone a colonoscopy or endoscopy.

Given the potential for antibiotic‐related effects on coagulation, particularly with PTZ, the infectious disease team was reconsulted. Antibiotic therapy was revised, and monotherapy with daptomycin was initiated to mitigate potential drug‐induced bleeding risks.

An urgent esophagogastroduodenoscopy (EGD) was performed. The stomach contained a large amount of clotted and fresh blood. After irrigation and suction, an actively oozing 6‐mm cratered duodenal ulcer (Forrest Ib) was identified in the second portion of the duodenum. Endoscopic hemostasis was successfully achieved as the primary intervention to stop the bleeding; this involved injection of 3 cc of epinephrine around the ulcer base for vasoconstriction, followed by the application of three hemoclips for definitive mechanical vessel closure. The duodenum also showed evidence of old blood, suggesting prior bleeding events. High‐dose intravenous pantoprazole therapy was initiated post‐procedure per the gastroenterology team’s recommendation to reduce the risk of rebleeding.

The clinical course was reflected in the serial laboratory trends. Upon admission (Day 1), all coagulation studies were within normal limits (platelets: 236 × 10^9^/L, PT: 11.8 s, INR: 1.1, and aPTT: 34 s). Coinciding with the massive GI bleed on Day 2, a marked decline in Hb was accompanied by a rise in PT to 13.6 s and INR to 1.3 and a significant acute‐phase rise in fibrinogen to 628 mg/dL. The aPTT increased slightly to 39 s. Notably, the platelet count remained stable within the normal range throughout the hospitalization (245 on Day 1, 266 on Day 2, 269 on Day 3, and 289 × 10^9^/L by Day 7), arguing against thrombocytopenia as a cause. Following the discontinuation of rivaroxaban and PTZ and after successful endoscopic hemostasis, the coagulation parameters progressively normalized and were within normal limits by Day 7. While rivaroxaban‐specific anti‐factor Xa levels were not obtained, the temporal pattern of PT/INR elevation and resolution aligns with the introduction and withdrawal of the suspected interacting drugs.

By the third hospital day, the patient demonstrated significant clinical improvement, marked by the cessation of bleeding episodes and stabilization of vital signs. Laboratory results showed favorable trends, including a rising Hb level, which stabilized after the endoscopic intervention. The patient was transitioned from the intensive care unit to a general medical floor and was able to resume oral intake without complications. On Day 6, following a shared decision‐making process with the patient who denied any further melena or abdominal pain, rivaroxaban was restarted.

## 3. Discussion

Here, we report a novel case of massive GI bleeding potentially linked to PTZ use in the context of concurrent rivaroxaban therapy. While specific laboratory investigations to confirm this association were not performed, the clinical presentation and supporting literature strongly suggest PTZ as a contributing factor. Managing antibiotic therapy alongside anticoagulants remains a complex challenge, especially in patients with cardiovascular comorbidities. PTZ, a broad‐spectrum antibiotic widely used for severe infections, is associated with an increased risk of bleeding [5–7] (Table 2). This risk appears to be amplified in patients receiving anticoagulants like rivaroxaban, as piperacillin, a key component of PTZ, has been shown to impair platelet aggregation, thereby exacerbating bleeding tendencies [6].

**TABLE 2:** Case reports of adverse reactions to piperacillin/tazobactam.

Case no.	Patient information	Condition/chief complaints	Drug involved	Adverse drug reaction	Diagnostic findings
1	55‐year‐old Vietnamese man, hypertensive [5]	Intracranial hemorrhage	Piperacillin–tazobactam	Abnormal platelet function assays (PFAs)	Abnormal PFAs noted after starting treatment
2	73‐year‐old man [6]	*Pseudomonas aeruginosa* ventilator‐associated pneumonia	Piperacillin–tazobactam	Severe pulmonary hemorrhage, bloody diarrhea	Normal platelet count and coagulation tests, impaired ADP‐dependent platelet aggregation
3	50‐year‐old woman, history of bronchial asthma [7]	Dry cough, breathlessness	Piperacillin–tazobactam	Severe immune thrombocytopenia resulting in epistaxis	Positive IgG and IgM antibodies specific for piperacillin against platelets, confirmed by immunoprecipitation and flow cytometry
4	52‐year‐old white male, history of deep vein thrombosis on rivaroxaban	Severe cellulitis, gastrointestinal bleeding	Piperacillin–tazobactam	Massive gastrointestinal bleeding shortly after drug initiation	Clinical observation and symptom onset post‐drug initiation

*Note:* This table summarizes four case reports detailing adverse reactions associated with the use of piperacillin/tazobactam. It includes patient demographics, clinical presentations, specific drug involvement, described adverse reactions, and diagnostic findings. Legends: Author names: full citation of the study author(s); patient information: age, sex, and relevant medical history; condition/chief complaints: primary condition or complaint upon presentation; drug involved: the medication suspected to cause the adverse reaction; adverse drug reaction: noted side effects or reactions to the drug; diagnostic findings: relevant clinical or laboratory findings related to the adverse drug reaction. ADP (adenosine diphosphate), IgG (immunoglobulin G), IgM (immunoglobulin M), and GI (gastrointestinal).

Abbreviations: PFAs, platelet function assays; RBC, red blood cell.

The rapid onset of massive GI bleeding merely one day after initiating PTZ raises a valid question regarding the primary etiology. We must consider the alternative explanation of a stress‐related mucosal injury, given the context of acute infection. However, several factors make this less likely as the sole cause. The patient lacked many high‐risk features for stress ulcers, such as mechanical ventilation, burns, central nervous system injury, or profound coagulopathy at presentation. His hemodynamic stability on admission further reduces this probability. Instead, we propose a synergistic model [11]. A preexisting, subclinical duodenal ulcer (of unknown etiology, potentially *H. pylori*) may have been present. The introduction of PTZ, known to cause immediate platelet dysfunction, in a patient already therapeutically anticoagulated with rivaroxaban, could have disrupted the delicate hemostatic balance at the ulcer site, transforming it from a silent lesion into a source of life‐threatening hemorrhage. The temporal relationship is consistent with a drug‐triggered event [6]. This hypothesis is supported by the normal platelet count, which rules out thrombocytopenia but is consistent with PTZ‐induced functional platelet impairment, and the only modest elevation in PT/INR, which indicates that a full vitamin K–deficient coagulopathy had not yet developed.

Moreover, it remains unclear whether the bleeding resulted from a direct interaction between anticoagulant and antibiotic [8, 12], or if synergistic effects from other concurrently administered antibiotics to treat extensive cellulitis contributed to the event [13]. While PTZ and rivaroxaban are likely contributing factors, it is important to consider the potential for a cumulative effect when multiple antibiotics are prescribed concurrently. Certain antibiotics, like cephalosporins or fluoroquinolones, have also been associated with coagulopathies or disturbances in platelet function [8, 12, 14]. Further investigation into the role of coadministered antibiotics in altering the coagulation cascade, either through direct interactions or via indirect mechanisms (such as gut flora disruption), is necessary [15].

We applied the Naranjo Adverse Drug Reaction Probability Scale to formally assess the association [16]. When scored strictly according to the validated criteria, the result is as follows: The adverse event appeared after the suspected drug (PTZ) was administered (+2). The bleeding improved after the drug was discontinued; however, definitive endoscopic hemostasis was the primary intervention, making it difficult to attribute improvement solely to drug cessation (0). The event was confirmed by objective evidence (endoscopic visualization of the ulcer and a sharp drop in Hb) (+1). Other potential causes were present, including a duodenal ulcer and the patient’s underlying rivaroxaban therapy (−1). The event was similar to known adverse reactions of the drug (PTZ‐associated coagulopathy) (+1). No evidence of the event appeared when the patient was not receiving the drug (0, as he was on rivaroxaban throughout). The event was confirmed by objective data (this is the same as point 3 and should not be double‐counted) (0). The event appeared after a rechallenge was performed (0, as rechallenge was not performed). When placed on a drug of similar potency, the patient did not experience a similar event (0, not assessed). This yields a total score of 3, which places this adverse reaction in the “Possible” category. This recalibrated assessment underscores that while a causal link is plausible given the temporal relationship, it cannot be definitively established in this single case, especially in the context of a confirmed anatomical bleeding source and concomitant anticoagulation.

Piperacillin’s ability to inhibit platelet function, although beneficial in preventing thrombosis, may inadvertently enhance the anticoagulant effect of rivaroxaban, a factor Xa inhibitor [5]. This potential interaction may be particularly significant given the absence of platelet count changes. In the presence of other factors like antibiotic therapy, unexpected changes in coagulation tests can occur. Such disturbances might suggest an interaction at the level of the coagulation cascade, potentially affecting factor X activity and thus potentiating the effect of rivaroxaban.

Additionally, the interaction of broad‐spectrum antibiotics with the vitamin K synthesis pathway is well documented [17, 18]. Antibiotics, particularly those that affect the gut flora like cephalosporins and PTZ, can lead to a deficiency in vitamin K, a critical cofactor for synthesizing clotting factors II, VII, IX, and X. The typical pathway through which rivaroxaban works—factor X inhibition—could be exaggerated by a concurrent decrease in vitamin K–dependent clotting factors, thereby increasing the risk of bleeding [14, 18].

Although rivaroxaban is known to affect INR [19], Thabit et al. reported that the interaction between PTZ and rivaroxaban might involve disruption of gut flora, impacting vitamin K production and increasing bleeding risk [14]. While certain cephalosporins can directly affect PT, it is unclear if PTZ has a similar effect [13]. Clinical studies on this specific interaction are limited, highlighting the need for further research, especially given the potential for serious complications like massive GI bleeding as observed in our case report.

This situation is complicated further in patients who, like the one discussed, present with infection and inflammation, which themselves can predispose to elevated INR levels [20, 21]. Infection and inflammation can both directly affect coagulation by increasing the levels of proinflammatory cytokines, which can alter coagulation profiles, including INR [22, 23]. Inflammatory responses are known to increase fibrinogen levels and reduce antithrombotic proteins, which, in conjunction with anticoagulant therapy, can disturb the delicate balance between clot formation and bleeding risk [22, 23].

The concomitant use of antibiotics and anticoagulants requires diligent monitoring. Clinically significant bleeding due to the interaction between PTZ and anticoagulants like rivaroxaban is relatively rare but significant enough to warrant close observation. This is especially pertinent during the initiation and discontinuation phases of antibiotic therapy, where abrupt changes in gut flora and vitamin K metabolism may lead to unanticipated shifts in coagulation status. This case underscores the importance of vigilant monitoring of coagulation parameters, particularly in patients with multiple bleeding risk factors. It also serves as a reminder to prioritize antibiotic stewardship whenever feasible [21, 24].

Notably, the interaction in our case differs from prior reports of bleeding events related to rivaroxaban use, as this patient exhibited no significant thrombocytopenia and no previous history of GI bleeding. This differentiates our case from those in which the bleeding risk is primarily linked to changes in platelet counts or preexisting bleeding disorders. Moreover, we speculate that the coadministration of antibiotics, such as PTZ, may disrupt the gut flora and lead to a vitamin K deficiency, which in turn exacerbates rivaroxaban’s anticoagulant effects. This potential cumulative effect—where both direct drug interactions and indirect disruptions (such as gut flora imbalance) contribute to the bleeding risk—has not been extensively explored in the literature, making this case especially noteworthy.

The interaction between rivaroxaban and PTZ is likely pharmacodynamic rather than pharmacokinetic. Rivaroxaban is metabolized via P‐glycoprotein and CYP3A4, pathways not significantly inhibited by PTZ, making a pharmacokinetic interaction unlikely [25]. Instead, their combination poses a synergistic risk: Rivaroxaban inhibits factor Xa (impairing coagulation) while PTZ independently disrupts platelet function and may reduce vitamin K production. This simultaneous attack on multiple hemostatic pathways significantly increases bleeding risk.

Our case report has several important limitations. As a single case report, it is inherently limited by the small sample size and cannot establish definitive causality. Most significantly, we lacked specific laboratory investigations to definitively confirm the pathophysiological mechanism of the proposed PTZ–rivaroxaban interaction. Specialized tests such as platelet function studies (e.g., light transmission aggregometry to demonstrate PTZ‐induced platelet dysfunction) or rivaroxaban‐calibrated anti‐factor Xa activity levels were not performed [26, 27]. In the acute setting of a massive, life‐threatening GI hemorrhage, the clinical focus justifiably shifts to immediate resuscitation, endoscopic hemostasis, and reversal of coagulopathy, rather than procuring specialized assays for adverse drug reaction confirmation, which are often not readily available. Consequently, our attribution of causality remains inferential, based on the compelling temporal relationship, exclusion of alternative causes, and biological plausibility drawn from the literature. While this precludes definitive proof, we believe this report serves a crucial role as a hypothesis‐generating signal. It highlights a potential clinical danger that warrants a higher index of suspicion among clinicians and provides a clear rationale for future research such as large‐scale pharmacoepidemiologic studies to quantify the bleeding risk and prospective trials incorporating platelet function tests and drug level monitoring to elucidate the precise pharmacodynamic and/or pharmacokinetic mechanisms.

This case prompts a broader discussion on the safety profiles of drugs like rivaroxaban when used in conjunction with other medications that can interfere with the coagulation cascade. In addition to the well‐known effects on clotting factor synthesis and platelet function, there may be other subtler interactions, such as changes in liver enzyme activity or altered drug metabolism, that contribute to these adverse events [4]. Given the potential for significant clinical implications, these interactions suggest a need for guidelines that specifically address the management of anticoagulation in patients receiving treatments that may disturb the hemostatic balance. These guidelines could include recommendations for more frequent monitoring of coagulation tests (e.g., INR and PT) during antibiotic therapy, tailored anticoagulation dosing regimens, or adjustments in vitamin K supplementation in high‐risk patients.

## 4. Conclusion

This potential case of drug interaction adds to the existing knowledge by highlighting several key aspects. First, it underscores the need for a high threshold in initiating strong antibiotics in patients on DOACs, particularly during stressful situations such as infections, to mitigate the potential bleeding risks. Second, it emphasizes the importance of antibiotic stewardship, particularly in the empiric use of multiple antibiotics such as PTZ, which may be excessive and inappropriate for uncomplicated cellulitis treatment. Lastly, this case report of a potential association contributes to the ongoing ambiguity in the literature regarding the exact mechanism behind coagulation abnormalities observed with PTZ. Although similar events have been reported previously, there remains a lack of laboratory‐based research exploring this phenomenon, further highlighting the need for future studies to clarify the underlying pathophysiology.

## Author Contributions

Abbas Mohammadi contributed to conceptualization, data curation, investigation, methodology, visualization, and both the original draft and review and editing of the writing. Masoud Bitarafan and AA contributed to data curation, supervision, validation, and both the original draft and review and editing of the writing. Masoud Bitarafan and Korosh Hamed Saedian contributed to investigation, methodology, and both the original draft and review and editing of the writing. Korosh Hamed Saedian contributed to both the original draft and review and editing of the writing.

## Funding

No funding was received for this manuscript.

## Disclosure

A preliminary version of this work was previously presented as an abstract at the American Heart Association (AHA) Scientific Sessions 2019. The abstract was published in Circulation (DOI: 10.1161/circ.150.suppl_1.4142117).

## Ethics Statement

Consent was obtained from the participant in this study.

## Conflicts of Interest

The authors declare no conflicts of interest.

## Data Availability

The data that support the findings of this study are available on request from the corresponding author. The data are not publicly available due to privacy or ethical restrictions.
